# Practice effects of personalized interventions with interdisciplinary teamwork in type 2 diabetes remission: a retrospective study

**DOI:** 10.3389/fendo.2024.1341531

**Published:** 2024-03-26

**Authors:** Xiaona Tian, Yujin Tang, Rongrui Hu, Jianhong Ye, Haixin Chen, Junjie Wu

**Affiliations:** ^1^ Eighth Clinical School, Guangzhou University of Chinese Medicine, Foshan, Guangdong, China; ^2^ Department of Endocrinology and Metabolism, Foshan Hospital of Traditional Chinese Medicine, Foshan, Guangdong, China; ^3^ Service Department, Guangzhou ShanMao Health Technology LTD, Guangzhou, Guangdong, China

**Keywords:** interdisciplinary, type 2 diabetes, personalized interventions, diabetes remission, retrospective study

## Abstract

**Objectives:**

A retrospective analysis of the clinical outcomes of personalized interventions for type 2 diabetes mellitus (T2DM) in an interdisciplinary team.

**Methods:**

Under the guidance of an interdisciplinary team, 40 patients with T2DM underwent a systematic examination at the beginning of the intervention, 3 months after the intervention, and 3 months of follow-up at the end of the intervention (i.e., at 6 months). Key indicators such as fasting plasma glucose (FPG), 2-hour postprandial glucose (2hPG), fasting insulin level (FINS), glycated hemoglobin (HbA1c), blood lipids, and body mass index (BMI) were measured.

**Results:**

After the 3-month intervention, participants’ BMI, FPG, 2hPG, FINS, and HbA1c improved significantly, with statistically significant differences (P<0.05).These metrics remained essentially stable at the 3-month follow-up. Of all the participants, 92.5% (37 cases in total) successfully discontinued their medication after 3 months of intervention, of which 80% (32 cases) remained stable during the 3-month follow-up after discontinuation, fulfilling the criteria for remission of T2DM; 2 cases successfully reduced the dose of their medication, and only 1 case was maintained on the original treatment.

**Conclusions:**

Through an interdisciplinary team intervention strategy, we significantly optimized the glucose metabolism, lipid metabolism, and BMI status of patients with T2DM, making diabetes remission an achievable goal, which provides valuable experience for further optimization of diabetes prevention and control protocols.

## Introduction

Type 2 Diabetes Mellitus (T2DM) was once viewed as a progressive, lifelong disease requiring medication (1–3). However, with a better understanding of diabetes and advances in the field of treatment, through intensive lifestyle interventions, medications, and metabolic surgeries, some diabetic patients have been able to completely discontinue their medications while keeping their blood glucose levels on target or within the normal range, achieving remission of their diabetes (4–7). This transformation not only significantly improves patients’ quality of life and reduces healthcare costs, but also helps to slow the progression of diabetes, which is important for preventing complications and improving disease prognosis.

The traditional management strategy for T2DM has been described as a “five-pronged chariot”, covering the five key aspects of diet, exercise, medication, blood glucose monitoring and health education (8, 9). However, in actual management, medication is still dominant. Guidelines increasingly emphasize the early use of more advanced and expensive hypoglycemic (10), lipid-lowering, and antihypertensive medications to control blood glucose and reduce associated complications and cardiovascular risk (2, 11, 12). In daily life, many T2DM patients lack adequate self-management, are more bound by traditional concepts, and tend to rely on medication for a long period of time, while ignoring the importance of comprehensive management.

Related research in 2011 showed that patients with T2DM could normalize beta cell function and hepatic insulin sensitivity by restricting dietary intake, which was associated with reduced triacylglycerol storage in the pancreas and liver. Since then, researchers have begun to delve into the possibility of disease reversal or remission (13). The line between mitigation and reversal is not always clear in this area. Reversal of T2DM refers to a change in the direction of the underlying pathogenic pathway that leads to a favorable recovery of the patient’s health. We believe that the term “remission” is more appropriate because it specifies that the patient’s metabolic status has improved to a nondiabetic level within the consensus definition of at least 6 months after cessation of glucose-lowering medication. However, it also means that there is a possibility of recurrence of the disease, rather than indicating that the disease has been completely and permanently cured (14). T2DM remission refers to a situation where, through specific interventions, a patient’s blood glucose can remain at target or normal levels without the use of glucose-lowering drugs (15). This shift not only signifies a more proactive treatment goal for patients but may also offer new strategies to alleviate pressures on the global health system. However, in the past few years, such studies have mainly focused on T2DM patients with concomitant obesity, metabolic surgery to achieve T2DM remission (16–18), or on a single area such as lifestyle (19), exercise, weight management, or insulin-intensive therapy to explore its effect on T2DM remission, which often focuses on adjusting for a specific factor and ignores the fact that T2DM is a complex metabolic disease caused by a combination of genetic and environmental factors T2DM is a complex metabolic disease with multiple risk factors resulting from a combination of long-term effects (20). Even in the United States, 15% of newly diagnosed T2DM patients have a body mass index (BMI) within the normal range, suggesting that remission of T2DM is not limited to those with a BMI of more than 25 kg/m^2^ (21–23). A comprehensive strategy is necessary to achieve diabetes remission, which requires the establishment of integrated teams for the multidimensional management of patients with T2DM. To the best of our knowledge, few studies have provided personalized and comprehensive interventions for patients with T2DM within the framework of interdisciplinary integrated team collaboration. Therefore, we conducted this study to focus on the effectiveness of a personalized and integrated approach to the practice of T2DM palliation under the guidance of an interdisciplinary team, to synthesize the existing evidence, and to provide directional recommendations for future research. We expect that this study will provide new perspectives and ideas for clinical practice, thereby advancing the progress of T2DM treatment strategies in a more beneficial and humane direction.

## Materials and methods

### Study design and participants

Inclusion criteria: (1) meeting the 1999 WHO diagnostic criteria for diabetes mellitus; (2) aged 18 -70 years; (3) insulin levels assessed by glucose tolerance test showing good pancreatic islet function; (4) normal cardiorespiratory fitness and absence of locomotor system disorders affecting exercise workouts; and (5) complying with the trial protocol and signing the informed consent.

Exclusion criteria: (1) adults with occult immune diabetes mellitus, type 1 diabetes mellitus and other types of diabetes mellitus; (2) those with acute complications of diabetes mellitus, such as ketoacidosis, lactic acidosis, and hyperosmolar hyperglycemic state; (3) those with acute and chronic metabolic acidosis, and acute infections; (4) women in pregnancy or breastfeeding; and (5) those with malignant tumors, and abnormalities of cardiac, renal, and hepatic functions. This study was reviewed and approved by the Ethics Committee of Foshan Hospital of Traditional Chinese Medicine (Ethics Approval Number: KY【2023】345).

Between March 2022 and March 2023, we conducted a comprehensive assessment of 56 patients diagnosed with type 2 diabetes mellitus in Foshan City Hospital of Traditional Chinese Medicine. Among them, 4 patients were unable to participate in this study due to insufficient fasting insulin level, 11 patients due to insufficient postprandial insulin, and 1 patient was unable to participate in this study due to both fasting and postprandial insulin insufficiency. The final 40 patients were managed with individualized and comprehensive diabetes treatment. We recorded the participants’ medication regimens in detail at the baseline assessment stage. Of these, eight patients were treated with a single hypoglycemic agent, including metformin, SGLT-2 inhibitors, and sulfonylureas. Seventeen patients were treated with a combination of two glucose-lowering agents, such as metformin with a sulfonylurea, or a sulfonylurea with a DPP-4 inhibitor or SGLT-2 inhibitor. Fifteen patients involved a combination of three different types of hypoglycemic agents, including different combinations of metformin, sulfonylureas, alpha-glucosidase inhibitors, SGLT-2 inhibitors, and DPP-4 inhibitors. None of the participants had received insulin therapy in the past three months. Before the intervention, the health manager had educated the patients about T2DM remission. The patients who participated in the study had carefully read and fully understood the diabetes remission program before the intervention.

### Type of intervention

The study’s intervention program integrated dietary modifications, exercise advice, health monitoring, medication use guidance, and psychological support to achieve a personalized diabetes mitigation strategy. Participants were enrolled in a 6-month telemanagement program consisting of dietitians, kinesiologists, endocrinologists, psychologists, and health administrators who provided ongoing, systematic services through a WeChat group. The specific responsibilities of each member in this study are detailed in **Table 1**. In this program, participants upload their diet, exercise, weight, blood glucose, and blood pressure data via WeChat on a daily basis. The remote management team would provide personalized advice to each participant based on these data, covering nutrition, exercise, and medication (24).

**Table 1:** Responsibilities of management team members.

Team members	Roles and responsibilities
Endocrinologist	Responsible for evaluating patients’ conditions, developing and adjusting medical regimens, and monitoring efficacy and safety. Introduces the process of medical program implementation and the mechanism and significance of T2DM remission to patients to ensure that they fully understand and actively cooperate with the treatment program.
Dietitian	Responsible for helping patients fully understand the medical nutrition treatment process, the division of labor of the collaborative team, and after assessment, develop a medical nutrition treatment plan for patients based on guidelines and evidence-based evidence, and continuously follow up on implementation to ensure that the treatment plan is effectively implemented.
Kinesiologist	In conjunction with the physician, provide patients with exercise prescriptions that are of interest to them, are easy to perform and can be consistently adhered to, and instruct patients in proper exercise skills and methods.
Health manager	To help patients understand disease-related knowledge, teach them self-management knowledge and skills, and enhance their implementation of and adherence to lifestyle interventions.
Counselor	Regularly communicate with patients to improve their confidence in treatment and reduce the impact of adverse emotions on treatment outcomes.

#### Dietary interventions

The dietary intervention consists of three main meals and three additional meals per day, taking into account adequate water intake and essential micronutrients. The three additional meals at each stage should be based on the principle of “no additional meal if they are not hungry”. To provide more personal flexibility, we allow patients to readjust their diets according to their individual preferences and needs, and provide professional guidance on diet and nutrition (25). Appropriate dietary and nutritional interventions are key to alleviating T2DM (26, 27). Measures such as the use of supplemental glycemic control foods and semi-replacement meals during treatment not only help to increase satiety, but also slow the absorption of carbohydrates, which in turn effectively aids in blood glucose management.

In our palliative practice, we implement an intensive dietary nutrition intervention program over a 4-12 week period. The program combines an energy-restricted balanced diet, a low-carb diet (28), ketogenic (29), intermittent fasting approach (30) and incorporates supplementation with key nutrients such as vitamin C, B vitamins, and micronutrients such as calcium and magnesium (31). During this process, we also meticulously managed and intervened in the patient’s appetite. We encourage our patients to slow down the speed of eating and increase the number of chews, chewing 20 to 40 times for each bite of food and pausing appropriately between meals. To further reduce the speed of eating, we recommend using non-dominantly hand-held chopsticks or eating with a fork, as well as decreasing the portion size of each bite of food. It is recommended to drink a moderate amount of water before meals and consume a small amount of nuts, such as 10 almonds or 20 peanuts. When it comes to the order of eating, soup is the first thing they drink at a meal to help create a feeling of fullness. Then eat vegetables and low-sugar fruits, which, because of their size and low energy, not only help to increase satiety, but also help to slow down the absorption of food. Next consume meat, which are relatively high in energy and can further increase satiety. Finally, moderate intake of staples and carbohydrates. They can be effective in stabilizing postprandial blood sugar because they are absorbed more slowly. It is also important to increase dietary fiber intake, such as by increasing the intake of foods such as oats, whole grain breads made from meal replacement flours with 80% of the starch removed, green leafy vegetables, and low-sugar fruits (32). This can significantly prolong the feeling of fullness due to its slow emptying rate in the stomach. During the fortification period, we recommend a low-carb diet ration of 25-45% carbohydrates, about 10-15% protein, and 40-60% fat intake. And in a long-term maintenance diet, the recommended percentage of carbohydrate intake is 50-55%, protein stays at 10-15%, and fat is 30-40% (33, 34).

#### Exercise interventions

The exercise physician will develop an exercise prescription for the patient. Exercise will be gradually increased from the first week of the intervention. The increase in exercise volume can be paused and enter a maintenance phase when the following criteria are met: a minimum of 150 minutes of moderate to high intensity aerobic exercise per week, each lasting 30 minutes, with exercise intervals of no more than two consecutive days, and moderate to high intensity resistance training three times per week (33). Depending on the individuality of the patient, the kinesiologist will decide on the timing of the increase in the amount of exercise and the selection of the appropriate exercise modality. The development of an individualized exercise program should, in principle, follow a gradual progression to ensure that the patient adapts gradually. All exercise programs are developed after evaluation by the kinesiologist.

#### Lifestyle and health monitoring

Adjust their routine to ensure that they go to bed on time and wake up early, aiming for 7-8 hours of adequate sleep each night. Increase daily activity and reduce sedentary behavior. To ensure accurate diabetes management, we continue to strengthen blood glucose monitoring and advise patients to measure fasting blood glucose at least once a day using a home glucose meter or ambulatory glucose meter. Every week, we choose a fixed time to measure body weight in the morning on an empty stomach and after completing a bowel movement. To ensure the accuracy of the measurement, it is recommended to do it under clothing of similar weight. In addition, waist circumference is measured once every two weeks in the morning while fasting and relaxed, using the belly button as a reference. Monitor blood pressure 1-2 times a week and try to do it at the same time in the morning. In addition, perform tests related to pancreatic function every three months.

#### Psychological interventions and medication guidance

Patients will receive regular follow-up visits from a counselor through a combination of online and offline visits. After a comprehensive assessment of the patient’s medical history, symptoms, FPG, 2hPG, HbA1c, pancreatic islet function, and lipids by an endocrinologist, the patient will be given the appropriate drug regimen for clinical treatment. Through dynamic observation of the above indicators, endocrine specialists will conduct one-on-one assessment of the patient to determine his/her medication strategy, including maintenance of current medication, reduction of dosage, or complete discontinuation of medication.

Discontinuation: Under expert supervision, when the patient’s condition has stabilized and FPG, 2hPG, and HbA1c have reached normal or personalized target levels and have been maintained for two weeks, the dosage of the medication will begin to be gradually reduced until it is discontinued. Even after stopping the medication, the patient still needs to receive ongoing interventions, testing and guidance.

### Follow-up program

We will conduct follow-up visits in the 1st, 2nd and 3rd month after the personalized intervention to ensure that patients maintain healthy lifestyle habits. If during the follow-up, we find that the patient’s blood glucose reaches the diagnostic criteria for T2DM, we will promptly advise them to go to the hospital to be evaluated by a professional doctor. Based on the assessment results, the doctor will decide on the appropriate treatment plan. If necessary, we can start this intervention program again. If medication is decided, we will record the name and dosage of the medication used by the patient. In addition, we also organize regular online and offline seminars on diabetes to help patients learn how to manage themselves.

### Observation indicators

The aim of this study was to assess the effectiveness of the intervention by comparing the patients’ BMI as well as the differences in glycemic and lipid biochemical indices before and after the intervention. The main blood glucose biochemical indicators we focused on were: FPG, 2hPG, HbA1c and fasting insulin level (FINS). And the insulin resistance index (HOMA-IR) was derived from the insulin calculation formula. Lipid biochemical indices included triglycerides (TG), total cholesterol (TC), high-density lipoprotein cholesterol (HDL-C) and low-density lipoprotein cholesterol (LDL-C).

Criteria for remission of type 2 diabetes mellitus were defined as an HbA1c value of less than 6.5% or an FBG of less than 7.0 mmol/L after at least 3 months of discontinuation of glucose-lowering medication or lifestyle intervention alone (14). According to Chinese standards, normal weight: 18.5 ≤BMI < 24kg/m^2^, overweight: 24≤ BMI < 28kg/m^2^, obese: BMI ≥ 28kg/m^2^.

### Statistical analysis

SPSS 26.0 statistical software was used for statistics and analysis. Normally distributed measurements were expressed as mean ± standard deviation (x^-^ ± s), and comparisons were made using the paired t-test. Non-normally distributed measures were expressed as *M(Q1, Q3)*, and comparisons were made using the *Kruskal-Wallis* rank sum test. Count data were expressed as constitutive ratios or rates (%), and comparisons were made using the χ2 test. Differences were considered statistically significant at *P*<0.05.

## Results

**Table 2** shows the basic characteristics of the patients before the intervention, out of these 40 participants, the mean age was 56.20 years and the mean BMI was 22.29 kg/m², there were 24 males and 16 females. The average duration of diabetes mellitus was 3.79 years, with 5 cases having a duration of 10 years or more, up to 16 years.

**Table 2:** Baseline characteristics of participants.

Characteristics	Participants (n = 40)
Male, n (%) Female, n (%)	24 (60) 16 (40)
Age,years (SD)	56.20 ± 7.76
Duration of T2DM,years (SD)	3.79 ± 4.04
Bodyweight, kg (SD)	63.22 ± 10.87
BMI,kg/m² (SD)	23.29 ± 2.88
Systolic Blood Pressure (mmHg) (SD)	125.35 ± 14.70
Diastolic Blood Pressure (mmHg) (SD)	78.00 ± 9.29

Data are in n (%); mean (SD); n, number.

### Change in weight and BMI

**Table 3** displays the changes in weight and BMI indices during the pre-intervention, post-intervention, and follow-up periods. One-way repeated measures a one-way repeated measures analysis of variance (ANOVA) was applied, as the weight and BMI data met the criteria of normal distribution and demonstrated sphericity (*Machly’s W* = 0.946, *p* = 0.349 > 0.05; *Machly’s W* = 0.953, *p* = 0.398 > 0.05). The analysis revealed significant differences in weight and BMI among the pre-intervention, mid-intervention, and post-intervention phases (*F*(2,78) = 50.86, *p* < 0.001; *F*(2,78) = 47.70, *p* < 0.001). Bonferroni multiple comparisons revealed that there was a significant difference in weight between post-intervention and follow-up compared to pre-intervention, with p-values < 0.05. When comparing post-intervention with follow-up, the p-value was > 0.05, indicating no statistical difference. In summary, there was a noticeable decrease in weight and BMI indices post-intervention. However, during the follow-up period, both weight and BMI remained stable, showing no further significant changes.

**Table 3:** Comparison of weight and BMI indicators.

	Baseline	3 months	6 months	F-value	P-value
Weight	63.22 ± 10.87	58.42 ± 10.29①	59.60 ± 10.43①	50.86	<0.001
BMI	23.29 ± 2.88	21.51 ± 2.62①	21.94 ± 2.62①	47.70	<0.001

Bonferroni post hoc tests were conducted to perform pairwise comparisons:

①indicates *P* < 0.05 compared to pre-intervention.

### Changes in blood glucose control levels

**Table 4** presents the changes in HbA1c, FPG, and 2-hour PG during the pre-intervention, post-intervention, and follow-up periods. Given the normal distribution of HbA1c, FPG, and 2-hour PG data, ANOVA was employed. HbA1c and FPG data satisfied the sphericity test (*Mauchly’s W* = 0.901, *P* = 0.138 > 0.05; *Mauchly’s W* = 0.974, *P* = 0.61 > 0.05), while 2-hour PG did not meet the assumption of sphericity (*Mauchly’s W* = 0.69, *P* = 0.01 < 0.05), and thus, Greenhouse-Geisser correction was applied. The analysis results indicated significant differences in HbA1c, FPG, and 2-hour PG levels among the pre-intervention, mid-intervention, and post-intervention phases (*F*(2,78) = 66.49, *P* < 0.001; *F*(2,78) = 32.35, *P* < 0.001; *F*(1.527, 59.55) = 22.87, *P* < 0.001). Bonferroni multiple comparisons revealed significant differences in HbA1c, FPG, and 2-hour PG levels between post-intervention and pre-intervention (*P* < 0.05) as well as between follow-up and pre-intervention (*P* < 0.05). However, no statistical differences were observed in HbA1c, FPG, and 2-hour PG levels between post-intervention and follow-up (*P* > 0.05). In summary, there was a significant decrease in HbA1c, FPG, and 2-hour PG indices after the intervention. However, during the follow-up period, both HbA1c, FPG, and 2-hour PG levels remained stable, showing no further significant changes.

**Table 4:** Comparison of glucose metabolism indicators.

	Baseline	3 months	6 months	F-value	P-value
HbA1c	7.13 ± 0.74	5.93 ± 0.47①	6.15 ± 0.64①	66.49	<0.001
FPG	7.42 ± 1.17	6.29 + 1.01①	6.20 ± 0.86①	32.35	<0.001
2hPG	13.54 ± 3.81	11.07 ± 3.29①	10.41 ± 2.44①	22.87	<0.001

① indicates *P* < 0.05 compared to pre-intervention.

### Changes in insulin resistance

**Table 5** illustrates the changes in FINS and HOMA-IR levels during the pre-intervention, post-intervention, and follow-up periods. Due to the normal distribution of FINS and HOMA-IR data, one-way repeated measures analysis of variance was employed. However, both FINS and HOMA-IR data did not meet the assumption of sphericity (*Mauchly’s W* = 0.826, *P* = 0.026 < 0.05; *Mauchly’s W* = 0.81, *P* = 0.018 < 0.05), necessitating the use of Greenhouse-Geisser correction. The analysis results indicated significant differences in FINS and HOMA-IR levels among the pre-intervention, mid-intervention, and post-intervention phases (*F*(1.70, 66.43) = 16.61, *P* < 0.001; *F*(1.68, 65.52) = 29.43, *P* < 0.001). Bonferroni multiple comparisons revealed significant differences in FINS and HOMA-IR levels between post-intervention and pre-intervention (*P* <0.05) as well as between follow-up and pre-intervention (*P* < 0.05). However, no statistical differences were observed in FINS and HOMA-IR levels between post-intervention and follow-up (*P* >0.05). In summary, there was a notable decrease in FINS and HOMA-IR indices after the intervention, while during the follow-up period, both FINS and HOMA-IR levels remained stable, showing no further significant changes.

**Table 5:** Changes in insulin resistance.

	Baseline	3 months	6 months	F-value	P-value
FINS	52.66 ± 21.36	40.72 ± 15.45①	40.56 ± 14.31①	16.61	<0.001
HOMA-IR	2.93 ± 1.34	1.94 ± 0.90①	1.89 ± 0.77①	29.43	<0.001

① indicates *P* < 0.05 compared to pre-intervention. The unit of FINS is pmol/L. (Same as **Table 8**). HOMA-IR=[FPG(mmol/L)×FINS(μU/mL)]/22.5, Insulin conversion factor: 1μU/mL≈6pmol/L (35).

### Lipid biochemical change

**Table 6** displays the changes in blood lipid biochemistry before and after the intervention. Comparison of the changes in blood lipid biochemistry, including TG, TC, HDL-C, and LDL-C, before and after the intervention was conducted using paired samples t-test, as the data met the criteria of normal distribution. The analysis results revealed that there were statistically significant differences (*P* < 0.05) in the mean values of TG, TC, and HDL-C between pre-intervention and post-intervention. The p-value for the mean LDL-C levels between pre-intervention and post-intervention was > 0.05, indicating no statistically significant difference.

**Table 6:** Comparison of indicators of lipid metabolism levels.

	TG	TC	HDL-C	LDL-C
Baseline	1.40 ± 0.70	5.01 ± 1.20	1.29 ± 0.27	3.34 ± 1.07
3 months	0.92 ± 0.41	4.54 ± 0.88	1.76 ± 0.40	3.11 ± 0.79
t-value	5.764	2.879	-8.751	1.597
P-value	<0.05	<0.05	<0.05	>0.05

### Comparison between remission and non-remission groups

**Table 7** shows the comparison of remission rates between males and females and remission rates under different menstrual phases in females. Corrected X^2^ test was used because n=40, 1≤T<5. Because X^2^ = 0.0, *P* = 1.00 > 0.05, not significant, there was no difference in T2DM remission by gender. In the female population, we conducted a comparative analysis of the effect of different menstrual stages on T2DM remission. Given the sample size of n=16, the *Fisher* X^2^ test was used in this study and the chi-square value (X^2^) = 1.11 was obtained while *P*=1.00 > 0.05, which is not significant. The insignificance of this result may stem from the small sample size of the study, which limits the ability to detect potential associations. Future studies could enhance the accuracy and reliability of statistical tests by increasing the sample size. **Table 8** shows the comparison of indicators between the remission and non-remission groups.

**Table 7:** Comparison of gender and rate of relief of menstrual status among women.

		Remission group	Non-remission group	χ2	P
Sex	Male	19 (79.2%)	5 (20.8%)	0.00	1.00
	Female	13 (81.3%)	3 (18.8%)		
	n	32	8		
Menstrual state	Menopausal	9 (81.8%)	2 (18.2%)	1.11	1.00
	Non-menopausal	4 (80.0%)	1 (20.0%)		
	n	13	3		

**Table 8:** Comparison of (mean standard deviation) between remission and non-remission groups.

	Age	Duration	Baseline					3 months					6 months				
			BMI	HbA1c	FPG	2hPG	FINS	BMI	HbA1c	FPG	2hPG	FINS	BMI	HbA1c	FPG	2hPG	FINS
Remission group	56.19± 7.85	2.66± 3.05	23.26± 3.07	7.09 ± 0.69	7.28± 1.15	13.65± 4.02	51.02± 22.19	21.51± 2.72	5.92 ± 0.43	6.15± 0.87	11.18± 3.47	39.91 ± 15.54	21.88± 2.75	5.92 ± 0.46	5.92± 0.72	10.54± 2.60	39.34± 13.69
Non-remission group	56.25± 7.92	8.29± 4.56	23.42± 2.09	7.28 ± 0.94	8.00± 1.13	13.08± 3.03	59.20± 17.35	21.50± 2.36	5.93 ± 0.64	6.86± 1.40	10.59± 2.60	43.93 ± 15.66	22.18± 2.19	7.07 ± 0.38	7.32± 0.33	9.92± 1.69	45.42± 16.63

We studied the remission of diabetes at different age of onset. The results of the study showed that in the age group of 30 to 40 years, 4 out of 6 participants succeeded in remission with a remission rate of 66.67%. Whereas in the age group of 40 to 50 years, 5 out of 8 participants had successful remission with a remission rate of 62.5%.In the age group of 50 to 60 years, 16 out of 18 participants had successful remission with a remission rate of 88.89%. In the age group of 60 to 70 years, 7 out of 8 participants were successfully relieved with a remission rate of 87.5%.When analyzing the overall effect of age of onset on remission in a comprehensive manner, we did not find a clear pattern. This phenomenon can be caused by a number of factors, including the length of time the patient has had the disease, adherence during treatment, and daily lifestyle. Meanwhile, the sample size of this study is limited, which may be a non-negligible factor affecting our conclusions. In future studies, we will expand the sample size to improve the statistical credibility of the data.

## Discussion

In recent years, the dramatic increase in type 2 diabetes globally has been largely attributed to environmental factors, with people’s poor lifestyles playing a key role. Although each patient needs to receive a basic treatment approach, in practice we still need to develop an individualized treatment plan based on each patient’s specific situation. This individualized approach takes into account the patient’s genetic background, lifestyle, metabolic status and differences in response to treatment. Recognizing this, our research team adopted an innovative strategy by assembling an integrated interdisciplinary team. The objective was to employ a personalized approach in combatting T2DM and to evaluate its clinical effectiveness. The study’s findings are promising. This comprehensive intervention approach not only significantly enhanced patients’ blood glucose control, rejuvenated pancreatic β-cell function, and improved insulin sensitivity, but also optimized BMI and lipid metabolism profiles. More importantly, patients in T2DM remission were able to cease using glucose-lowering medications. This not only alleviated their psychological stress but also bolstered their confidence in leading a healthier life. As a result, patients experienced an improved quality of life, delayed disease progression, and a reduced risk of complications. This provides new ideas for the future prevention, control and treatment of T2DM. In a reversal of previous perceptions, T2DM is not necessarily a progressive disease. It is possible to achieve remission through personalized intervention. This brings a challenge to the long-held notion that T2DM patients need to take medication for life.

In this study, which focused on the middle-aged and elderly population, At the end of the study, 37 (92.5%) of all participants successfully discontinued their medication after the intervention; two others failed to completely discontinue the medication but succeeded in reducing the dosage, and only one continued with the original medication. Notably, 32 (80%) of the patients who successfully discontinued the drug remained in stable status 3 months after discontinuation, fulfilling the remission criteria for T2DM.For those five patients who failed to achieve sustained stabilization, there may be multiple reasons. First, older age and longer duration of diabetes were key predictors of failure to achieve remission. Patients who have had diabetes for more than 10 years have a significantly reduced chance of remission. Reduced insulin sensitivity and decreased insulin secretion with advancing age made the pre-intervention status of these patients even more unfavorable, making it predictably less likely that they would go into remission. Patients who have had diabetes for more than 10 years have a significantly reduced chance of remission. Reduced insulin sensitivity and decreased insulin secretion with age make the pre-intervention status of these patients even more unfavorable, making it predictably less likely that they will go into remission. Further, patient compliance is extremely critical. If they don’t follow their doctor’s instructions to the letter after stopping their medication, or fail to adhere to recommended lifestyle and dietary habits, or don’t engage in regular glucose monitoring, they may be at an increased risk of relapse. It is worth pointing out that even among patients who did not fully meet the remission criteria, they achieved significant improvements in glucose metabolism. The trends in lipid biochemistry changes before and after the intervention as a whole showed a significant improvement.

In our study, the mean value of baseline BMI was 23.29 ± 2.88. Out of 40 study subjects, 14 were categorized as overweight and 3 of them met the criteria for obesity. Surprisingly, however, despite the fact that 65% of the participants in the study were categorized as not being in the overweight or obese group, these individuals demonstrated a considerably higher rate of T2DM remission. This observation can be linked to the personal fat threshold (PFT) hypothesis. According to this hypothesis, the storage capacity of subcutaneous fat may vary from individual to individual. The theory is that each individual may have a unique PFT, a threshold that determines their susceptibility to developing T2DM (21). T2DM may develop when an individual gains enough weight to exceed their PFT, as this fat accumulation may lead to insulin resistance and metabolic problems. However, by losing weight and returning to a weight below the personal adiposity threshold, normal blood glucose levels are expected to be restored, i.e., remission of T2DM is achieved (22). This perspective is important because it suggests that the disease is not a weight-dependent manifestation of different pathogenic mechanisms, but rather that there are common physiologic mechanisms. It also provides a more personalized approach for non-overweight/obese T2DM patients in order to develop more effective strategies based on each individual’s physiology.

Results from The Diabetes Remission Clinical Trial (DiRECT) showed that at 1-year follow-up, 46% of patients achieved remission of T2DM through intensive dietary and lifestyle interventions, compared with only 4.0% of patients in the control group. At the end of RCT, the 2-year intention to treat remission rate was 36.0% (15, 36). Taheri, S et al. randomly assigned T2DM patients with disease duration of up to 3 years in a 1:1 ratio to an intensive lifestyle intervention group and a control group with usual medical care. After a 1-year follow-up, the results showed that the remission rate of T2DM was as high as 61% in the intervention group, whereas in the control group, the remission rate was only 12% (37). In comparison, the intervention approach used in this study demonstrated a more significant mitigating effect. This may be attributed to our interdisciplinary synergy, personalized and comprehensive intervention strategies, well-targeted education and guidance, and comprehensive and multidimensional health management of patients. However, it is important to note that our follow-up period was relatively short. This may mean that the effectiveness and persistence of the intervention may change as time advances. The higher remission rate in this study may be related to the relatively short follow-up period, whereas longer follow-up periods have resulted in lower remission rates in other studies. This study shows data from 6 months of systemic management. Further systemic management is needed for these participants, and we will conduct a long-term follow-up study and publish long-term findings in the future.

Our treatment program does require an increased professional time commitment from participants in the short term compared to traditional clinical treatment. However, our program introduces an element of self-management and collaboration that consciously reduces the burden on patients with the help of modern technology and remote support. Patients use WeChat to clock in and out, participate in health group interactions, and follow professional advice on exercise and diet management. This flexible teletherapy approach not only reduces the amount of time patients spend traveling to the hospital, but also improves the efficiency of treatment. In the long run, we see this investment of specialized time as a great long-term investment. By engaging patients to better understand their health status and actively participate in their treatment plans, we anticipate that treatment outcomes will be improved, resulting in a reduction in the risk of complications and frequency of medical visits. On the issue of sustainability, we emphasized the need to work closely with patients to develop practical treatment plans to reduce their burden. At the same time, the support of the healthcare system and the allocation of resources are key to ensuring long-term sustainability. By continuously improving and optimizing treatment processes, we expect to achieve a sustainable balance between the time investment of patients and professionals.

There are some limitations to this study. First, there was no blank control group, which may have had some impact on the results. Second, some of the results may be biased due to the limited sample size. Third, there may be some bias in participants’ self-reported data on dietary intake and exercise. In order to more accurately assess the effects of personalized comprehensive interventions in T2DM remission, we suggest future randomized controlled studies covering multiple centers with larger sample sizes, higher quality, and longer follow-up periods. This will not only help validate our findings, but also further refine and optimize treatment strategies for T2DM.

## Data availability statement

The original contributions presented in the study are included in the article/supplementary material. Further inquiries can be directed to the corresponding author.

## Ethics statement

The studies involving humans were approved by Foshan Hospital of Traditional Chinese Medicine Medical Ethics Committee. The studies were conducted in accordance with the local legislation and institutional requirements. The participants provided their written informed consent to participate in this study.

## Author contributions

XT: Conceptualization, Writing – original draft. YT: Writing – review & editing. RH: Writing – original draft, Data curation, Software. JY: Conceptualization, Writing – review & editing. HC: Data curation, Writing – review & editing. JW: Data curation, Writing – review & editing.
